# Implantation of Neuronal Stem Cells Enhances Object Recognition without Increasing Neurogenesis after Lateral Fluid Percussion Injury in Mice

**DOI:** 10.1155/2018/4209821

**Published:** 2018-02-06

**Authors:** Laura B. Ngwenya, Sarmistha Mazumder, Zachary R. Porter, Amy Minnema, Duane J. Oswald, H. Francis Farhadi

**Affiliations:** ^1^Department of Neurological Surgery, The Ohio State University Wexner Medical Center, 410 W 10th Avenue, N1021 Doan Hall, Columbus, OH 43210, USA; ^2^Department of Neurosurgery, University of Cincinnati, 231 Albert Sabin Way, MSB 5209, ML 0517, Cincinnati, OH 45267, USA

## Abstract

Cognitive deficits after traumatic brain injury (TBI) are debilitating and contribute to the morbidity and loss of productivity of over 10 million people worldwide. Cell transplantation has been linked to enhanced cognitive function after experimental traumatic brain injury, yet the mechanism of recovery is poorly understood. Since the hippocampus is a critical structure for learning and memory, supports adult neurogenesis, and is particularly vulnerable after TBI, we hypothesized that stem cell transplantation after TBI enhances cognitive recovery by modulation of endogenous hippocampal neurogenesis. We performed lateral fluid percussion injury (LFPI) in adult mice and transplanted embryonic stem cell-derived neural progenitor cells (NPC). Our data confirm an injury-induced cognitive deficit in novel object recognition, a hippocampal-dependent learning task, which is reversed one week after NPC transplantation. While LFPI alone promotes hippocampal neurogenesis, as revealed by doublecortin immunolabeling of immature neurons, subsequent NPC transplantation prevents increased neurogenesis and is not associated with morphological maturation of endogenous injury-induced immature neurons. Thus, NPC transplantation enhances cognitive recovery early after LFPI without a concomitant increase in neuron numbers or maturation.

## 1. Introduction

Annually, over 2.8 million Americans experience a traumatic brain injury (TBI), and over 10 million people worldwide are affected [1–4]. TBI causes significant morbidity and mortality, and despite the financial and social burden on society, there has yet to be a successful therapeutic intervention for TBI. Cognitive deficits after TBI are debilitating. Patients with cognitive deficits are often unable to return to work and have reduced productivity in society. The more we understand about TBI-induced cognitive deficits, and ways to treat them, the better we can reduce the societal impact of TBI.

Years of research in TBI have resulted in many well-defined animal models, yet cognitive deficits after TBI persist, and proven interventions for cognitive recovery are lacking. Animal studies have shown that the transplantation of stem cells shows promise for the recovery of cognitive function after experimental TBI [5–8]. Studies have also shown that priming of the environment, and secretion of growth factors can facilitate graft survival and integration [9–12], but the mechanism by which stem cell transplantation mediates improvement in cognitive function after experimental TBI is poorly understood.

The hippocampus is a critical structure in learning and memory, is particularly vulnerable after TBI [13, 14], and is the potential site wherein transplanted stem cells mediate cognitive improvement after TBI. Within the hippocampal formation, the dentate gyrus plays a special role: it sits at the beginning of a trisynaptic circuit of memory formation, is necessary for encoding multiple inputs for contextual pattern separation [15–17], and supports adult neurogenesis. Neurogenesis, the production of new neurons from endogenous stem cells, has been demonstrated in a wide range of species including rodents, nonhuman primates, and humans [5, 9, 18–20]. After TBI, there is an increase in hippocampal neurogenesis [21–26]. Complete disruption of adult neurogenesis impairs the ability for cognitive recovery after TBI [27, 28]. Yet, after TBI, there is also cell death in the hippocampal dentate gyrus, and the process of adult neurogenesis is disrupted [29]. Thus, the role and integration of adult-generated neurons in the setting of TBI and recovery require further study.

Because of the important role of the hippocampus and neurogenesis in cognition, we hypothesized that the mechanism by which stem cell treatment improves cognitive recovery after TBI involves a modulation of endogenous hippocampal neurogenesis. To evaluate this, we performed lateral fluid percussion injuries in adult mice and transplanted neural progenitor cells into the vicinity of the hippocampal formation after injury. Here, we show that neural progenitor cell transplantation enhances cognitive function without an associated increase in endogenous neurogenesis.

## 2. Materials and Methods

### 2.1. Animal Model of Experimental TBI and Surgical Procedures

We used a total of 118 adult C57BL/6 mice for this study, of which 64 animals were not excluded following the criteria below and were therefore included in the final analysis. All C57BL/6 mice originated directly from Charles River and were maintained on a pure genetic background in our internal colony. Animals were housed in a 12/12 light-dark cycle with food and water *ad lib*. All animal protocols were approved by The Ohio State University's Institutional Animal Care and Use Committee and were in accordance with the Guide for the Care and Use of Laboratory Animals [30].

Animals underwent a lateral fluid percussion injury (LFPI) similar to that previously described [30–32]. Briefly, animals were anesthetized using an intraperitoneal injection of ketamine and xylazine. Once a surgical plane of anesthesia was induced, verified by decreased respiratory rate and lack of response to toe pinch, animals were placed on a heating pad set at 37°C and secured in a stereotaxic head holder (Stoelting, Wood Dale, IL, USA). A midline incision was fashioned, the skin was retracted, and the skull exposed. A craniectomy, centered at 2 mm posterior to bregma and 2 mm right of midline, was fashioned using a 3 mm outer diameter trephine, and a rigid hub was secured to the skull. Any animal noted to have a dural breach was euthanized and excluded from the study (*n* = 8).

Animals were recovered from anesthesia, ambulating, and interacting appropriately prior to LFPI. Four to eight hours after surgery, animals were briefly anesthetized in a chamber consisting of 5% isoflurane for three minutes in preparation for injury induction. Animals were observed for signs of immobility and decreased respiratory rate. Once anesthetized, animals were connected to the fluid percussion device (Custom Design and Fabrication, Richmond, VA, USA) and a fluid pulse was delivered via release of the pendulum. Sham animals received a craniectomy, isoflurane anesthesia, and were connected to the device but did not receive the fluid pulse.

Each animal was disconnected from the device and immediately turned on its back. The amount of time, in seconds, that the animal required to spontaneously right itself was recorded as the righting reflex time (RRT). The RRT was used to confirm injury severity. All animals with a moderate-to-severe injury (defined as RRT between 260 and 660) were included in the study; injured animals with a RRT outside of this range were excluded (*n* = 19). Animals that developed a large brain herniation or expired in this immediate postinjury period were also excluded from the study (*n* = 24). An additional nine control animals that did not receive surgery were used as baselines for behavioral testing.

One week postinjury, injured animals were randomly assigned to a treatment group. Under ketamine/xylazine anesthesia, the previous craniotomy site was opened under sterile conditions. A microinjection pump system (Nanoliter 2010, World Precision Instruments, Sarasota, FL, USA) with motorized stereotactic control was used to target the hippocampal fissure at −2.2 mm anteroposterior, 1.4 mm mediolateral, and a depth of −2.0 mm in relation to bregma [33]. A freshly pulled glass pipette of 90–110 *μ*m diameter was used for each injection. A suspension of 100,000 embryonic stem cell-derived neural progenitor cells (NPC) in 1 *μ*L of vehicle, or vehicle alone, was injected at a rate of 46 nL/s. Injection time spanned three minutes, followed by a slow withdrawal of the pipette over 1.5 minutes. Animals with histological evidence of NPCs located cortically or within the ventricular system were excluded from the study (*n* = 3).

### 2.2. Preparation of Embryonic Stem Cell-Derived Neural Progenitor Cells

GFP-tagged embryonic stem cells (ES) derived from C57BL/6 mice were purchased (Gibco, ThermoFisher Scientific, Waltham, MA, USA). ES cells were cultured in knockout ES media (Gibco) containing 1400 U/mL of leukemia inhibitory factor (Millipore, Billerica, MA, USA) and passaged as previously described [34]. Within six passages of purchase, ES cells were used for differentiation to nestin-positive NPCs, as described [35, 36]. Briefly, ES cells were cultured in feeder-free conditions to allow formation of embryoid bodies in suspension. On day 4, embryoid bodies were collected from suspension, adhered onto tissue culture plates, and then cultured for 7 days in a medium of DMEM/F12 (Gibco) containing insulin (5 *μ*g/mL, Sigma-Aldrich, St. Louis, MO, USA), transferrin (50 *μ*g/mL, Sigma-Aldrich), selenium (30 nM, Sigma-Aldrich), and fibronectin (5 *μ*g/mL, R&D Systems, Minneapolis, MN, USA). Cells were then dissociated and replated onto polyornithine- (20 *μ*g/mL, Sigma-Aldrich) and laminin- (5 *μ*g/mL, ThermoFisher Scientific) coated plates. Cells were expanded in DMEM/F12-containing insulin (25 *μ*g/mL, Sigma-Aldrich), transferrin (50 *μ*g/mL, Sigma-Aldrich), selenium (30 nM, Sigma-Aldrich), progesterone (20 nM, Sigma-Aldrich), putrescine (100 *μ*M, Sigma-Aldrich), bFGF (5 ng/mL, Peprotech, Rocky Hill, NJ, USA), and laminin (5 *μ*g/mL, ThermoFisher Scientific). Cells were used for transplantation within 7 days of expansion in this medium. Prior to transplantation, cells were checked for viability with trypan blue and demonstrated an average of 93% viability. Cells were suspended in vehicle on ice during the duration of the surgery for transplantation. At the completion of the transplantation surgery, 80% of cells remained viable.

For immunocytochemistry, cells grown in parallel in slide-wells were fixed in cold 4% paraformaldehyde for 30 minutes. Slides were rinsed, blocked in 10% normal goat serum (Gibco, ThermoFisher Scientific) and incubated in primary antibody overnight (rat anti-nestin, 1 : 200, Abcam, Cambridge, MA, USA; rabbit anti-GFAP, 1 : 500, Dako, Agilent, Santa Clara, CA, USA; or goat anti-DCX 1 : 500, Santa Cruz, Dallas, TX, USA). The following day, cells were rinsed and incubated in secondary antibodies conjugated to Alexa Fluor 488 or 594 (1 : 250, ThermoFisher Scientific) for 2 hours at room temperature in the dark. After rinsing, slides were counterstained with DAPI (Sigma-Aldrich) and coverslipped with polyvinyl alcohol with DABCO antifading reagent (PVA-DABCO; Sigma-Aldrich). Analysis of cell phenotype was done using an Olympus confocal microscope. Cells used for transplantation were >80% nestin-positive, consistent with a neural progenitor cell phenotype.

### 2.3. ES-NPC Signature Quantitative Real-Time Polymerase Chain Reactions (qRT-PCR)

RNA was extracted from adherent ES cultures and from NPCs just prior to transplantation. RNA was extracted using the RNeasy Kit (Qiagen, Valencia, CA, USA), followed by on-column DNAse digestion using RNAse Free DNAse Kit (Qiagen). Four *μ*g of cDNA per replicate was prepared using Qiagen RT2 First Strand Kit or maloney's murine leukemia virus (MMLV) reverse transcriptase (Invitrogen, Carlsbad, CA). The cDNA was diluted in PowerUP SYBR green master mix (ABI, ThermoFisher Scientific) at a concentration of 100 ng/well and aliquoted into custom signature qRT-PCR array (Qiagen) 96-well plates, consisting of 27 stem cell, glial, and neuronal differentiation genes in triplicate. Relative gene expression was quantified using the ΔΔC_T_ method, with the significance threshold established at ±2-fold expression. Statistical significance was determined by calculating individual Student's *t*-tests by comparing within the control ES signature genes and between the NPC versus ES ΔΔC_T_ values.

### 2.4. Tissue Harvest and Histology

**Figure 1:**
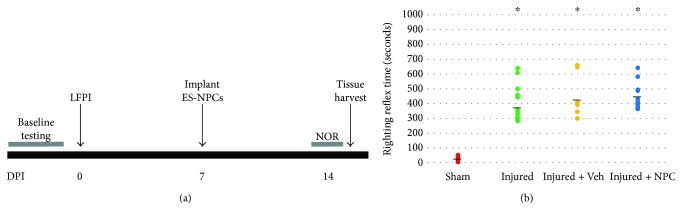
LFPI creates reproducible injuries with similar RRT among injured animal groups. (a) Timeline demonstrating the experimental design. Animals received acclimation and baseline behavioral testing prior to lateral fluid percussion injury (LFPI). Embryonic stem cell-derived neural progenitor cells (ES-NPCs) were implanted at 7DPI. One week after cell transplantation animals were tested on a hippocampal-dependent task, novel object recognition (NOR), and euthanized the following day. (b) Moderate-to-severe injuries were created using the FPI device, with injured animals showing RRT significantly greater than sham animals (^∗^*p* < 0.001 versus sham). Injured animals that were randomly selected to receive injections of vehicle or NPC did not show significantly different RRT from other injured animals (all *p* > 0.1). Bold lines represent the group mean values.

At one week postinjection, 15 days postinjury (DPI) (Figure 1(a)), animals were anesthetized with an intraperitoneal injection of ketamine and xylazine and then transcardially perfused with 0.1 M phosphate-buffered solution (PBS) followed by 4% paraformaldehyde. Brains were removed, postfixed in 4% paraformaldehyde overnight at 4°C, rinsed in PBS, and transferred to 30% sucrose for three days at 4°C. Brains were embedded in a cryomold using optimal cutting temperature compound (ThermoFisher Scientific), quickly frozen using dry ice, and stored at −80°C. Brains were sectioned in the coronal plane at 12 *μ*m thickness using a cryostat, collected in 10 evenly spaced series onto superfrost slides (ThermoFisher Scientific), and stored at −20°C until used for staining.

Histological confirmation of NPC transplantation was done utilizing the GFP positivity of the ES-NPCs. Green GFP fluorescence was directly visualized using epifluorescence or amplification of the GFP signal via immunohistochemistry to GFP. Sections were incubated with chicken anti-GFP (1 : 200, ThermoFisher Scientific) for 24 hours at 4°C followed by washes and incubation in biotinylated anti-chicken secondary antibody (1 : 200, Aves, Tigard, OR, USA). Antibody reactivity was amplified utilizing the ABC Elite kit (Vector Labs, Burlingame, CA, USA), and chromagen stained with DAB (Vector Labs). Sections were counterstained with 0.1% cresyl violet (Scy-Tek, Logan, UT, USA).

Evidence of new neuron generation was evaluated at 15 days postinjury (DPI) by immunohistochemical staining for the immature neuron marker doublecortin (DCX) [37]. Evenly spaced sections through the entire rostrocaudal extent of the hippocampal dentate gyrus were incubated overnight at 4°C in goat anti-doublecortin primary antibody (1 : 200, Santa Cruz) diluted in 3% horse serum (Gibco, ThermoFisher Scientific) and 0.3% triton-x (Sigma-Aldrich) in PBS. Following washes, sections were incubated in biotinylated horse anti-goat secondary antibody (Vector Labs). Similar to the above, sections were then incubated with ABC (Vector Labs), stained with DAB, and counterstained with 0.1% cresyl violet (Scy-Tek). Slides were blinded, and an unbiased stereological count of the estimated total number of DCX-positive neurons in the dentate gyrus granule cell layer was performed. For this analysis, the GCL was outlined at low magnification using a Nikon eclipse microscope and motorized stage. DCX-positive somas were counted at high magnification following the optical fractionator method [5, 38, 39] utilizing Stereo Investigator Software (MicroBrightField Bioscience, Williston, VT, USA). DCX-positive cells within the GCL were counted using a grid size of 75 *μ*m × 75 *μ*m and a counting frame of 50 *μ*m × 50 *μ*m on every 20th evenly spaced section through the dentate gyrus. DCX-positive cells within the hilus were counted using an exhaustive counting scheme on every 20th section through the dentate gyrus. Due to the thinness of the sections used, we utilized a bottom *z*-axis exclusionary plane to avoid overcounting bias.

For analysis of dendrite length, two randomly selected brains per group were cut at 60 *μ*m and collected free-floating in PBS. Tissue was then processed free-floating using immunofluorescence techniques for DCX. Sections were incubated in goat anti-doublecortin (1 : 50, Santa Cruz) overnight at room temperature, followed by washes in PBS and incubation in Alexa Fluor 594 conjugated donkey anti-goat (1 : 200, Life Technologies) for 3 hrs at room temperature in the dark. Sections were counterstained with DAPI (Sigma-Aldrich) and wet-mounted. Sections were coded and then analyzed using an Olympus FV100 confocal microscope. Z-stack pictures were taken throughout the entirety of the section and saved for offline analysis. Per animal, the five most mature-appearing cells with complete dendritic trees within the section were chosen for analysis. Dendritic length and branching pattern were calculated using the Sholl analysis plugin algorithm for ImageJ/Fiji [40–42].

### 2.5. Behavioral Studies

Prior to inclusion in the study, animals were acclimated to the housing and behavioral testing environment for a minimum of three days. Cognitive testing was performed at 14 DPI using the novel object recognition (NOR) task [43–45]. All objects used were of similar size and complexity and were tested for equal preference and lack of avoidance. One day prior to testing, animals were acclimated to the activity box open-field area (Accuscan, Omnitech, Columbus, OH, USA) for 10 minutes. On the day of testing, animals were individually tested in the box for 10 minutes with two identical objects placed in the opposite corners of the 40 cm × 40 cm box. Beam breaks were recorded in the 10 cm × 10 cm zone surrounding the center of each object. During this task, zone activity was analyzed using Fusion software (Omnitech) to detect side preference or significant object avoidance. Additionally, the total exploration time of similar objects was recorded. Animals were returned to their home cage for a 1-hour interval. After this interval, animals were placed in the box with a familiar object and a novel object for five minutes. Object type and position were randomized across all groups. Objects and the activity box were cleaned between every trial to minimize olfactory cues. Testing sessions were videotaped for offline analysis. A single researcher blinded to the animal group performed the analysis. The amount of time the animal spent exploring each object was obtained by analysis of the video by a blinded observer. Object exploration was defined as the animal facing, sniffing, and otherwise interacting within 2 cm of the object. Grooming near an object, climbing on an object for extended periods, or back facing the object was not included as exploration time. Recognition of the novel object was calculated as a novel object discrimination ratio: (time-exploring novel object)/(time exploring familiar object + time exploring novel object). A ratio of 0.5 represents equal time with both objects, whereas a value above 0.5 demonstrates time spent preferentially with the novel object.

### 2.6. Statistical Analysis

Data analysis was done using SPSS statistics for Macintosh (v23, IBM). All data were tested for the assumptions of normality (Shapiro-Wilk test) and homogeneity of variances (Levene's test). Where all assumptions were met, between-group differences were analyzed with analysis of variance (ANOVA) and Tukey HSD post hoc analyses. Where the assumptions were not met, data was analyzed with the nonparametric Kruskal-Wallis test for independent samples. For NOR, each group was compared to the equal object exploration value of 0.5 via a one-sample *t*-test. A *p* value of 0.05 was used to indicate significance for all statistical tests.

## 3. Results

### 3.1. Lateral Fluid Percussion Injury Creates Reproducible Injuries

All injured animal groups differed significantly from shams (*n* = 14) in RRT (*χ*^2^[3] = 34.66, *p* < 0.001; Figure 1(b)). Kruskal-Wallis with pairwise comparisons shows significant differences versus sham (injured, *n* = 19; injured + vehicle, *n* = 9; injured + NPC, *n* = 13; all *p* < 0.001). There were no between-group differences among injured animals (all *p* > 0.1).

### 3.2. Transplanted Cells Differ in Immunocytochemical and Genetic Phenotype from Embryonic Stem Cells

**Figure 2:**
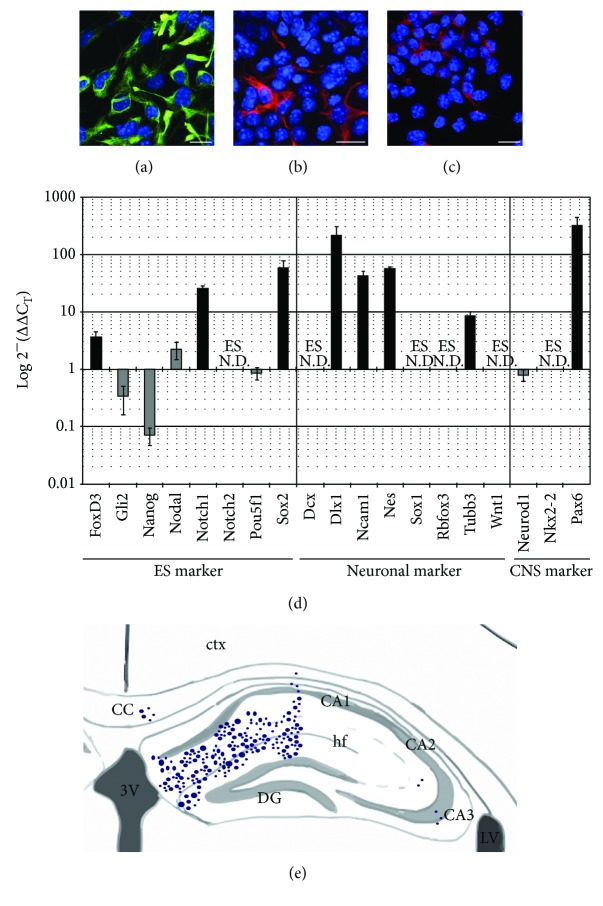
Embryonic stem cell-derived neural progenitor cells demonstrate mixed neuronal and glial features. (a) The majority of implanted cells were nestin-positive (green nestin antibody reactivity; blue = DAPI nuclear stain). A small proportion of cells demonstrated (b) GFAP and (c) DCX positivity (red = GFAP or DCX antibody reactivity, resp., blue = DAPI nuclear stain; scale bars = 20 *μ*m). (d) qRT-PCR was performed using 100 ng/reaction cDNA reverse transcribed from mRNA extracted from undifferentiated ES cultures (*n* = 4) and differentiated NPC cultures (*n* = 3). Twenty-seven signature marker genes were analyzed using the ΔΔC_T_ method. Statistical significance of genes that were shown to be 2-fold upregulated or downregulated in NPCs versus ES was determined using Student's *t*-test. Statistically upregulated genes (ΔΔC_T_ ≥ 2.0 and *p* ≤ 0.05) are shown in black. Statistically downregulated genes (ΔΔC_T_ ≤ 0.5 and *p* ≤ 0.05) are shown in dark gray. Light gray bars show statistically insignificant expression. ES N.D. = not detected in ES culture qRT reactions. Error bars indicate SEM. (e) Schematic demonstrating density and location of implanted cells one week posttransplantation. Data represents pooled results from all 12 animals that received NPCs (CA1, CA2, CA3 = hippocampal subfields Cornu Ammonis 1, 2, and 3; cc = corpus callosum; ctx = cortex; DG = dentate gyrus; hf = hippocampal fissure; LV = lateral ventricle; 3 V = third ventricle).

Embryonic stem cell-derived NPCs were characterized by immunocytochemical and genetic methods prior to transplantation. Immunocytochemistry confirmed a predominance of nestin positivity, with some GFAP and DCX immunoreactivity (Figures 2(a)–2(c)). To further confirm the transition from undifferentiated ES cells to differentiated NPCs, we performed qRT-PCR using RNA extracted from three independent cultures of each cell-type. The prepared cDNA was probed with eight ES-specific primers, eleven neuronal/central nervous system-specific primers (Figure 2(d)), and six glial-specific primers (data not shown). Overall, the neuronal probe's ΔΔC_T_ values showed that neuron-specific gene products were significantly upregulated or were not detected, in the NPCs versus the undifferentiated ES cells. Of note, *Ncam1* (ΔΔC_T_ = 42.8 ± 9.2, *p* = 0.0006) and Nestin (*Nes*, ΔΔC_T_ = 58.1 ± 4.2, *p* = 9.3 × 10^−11^) were significantly upregulated. In addition, doublecortin (*Dcx*), an immature neuronal microtubule-associated protein was not detected in the triplicate ES cultures but was highly expressed (avg. ΔC_T_ = 7.2) in NPCs. Of the well-established ES-specific gene products, *Gli2* (ΔΔC_T_ = 0.3 ± 0.2, *p* = 0.005) and *Nanog* (ΔΔC_T_ = 0.1 ± 0.02, *p* = 0.01) were significantly downregulated in the NPC cultures, and *Nodal* (ΔΔC_T_ = 2.2 ± 0.7, *p* = 0.19) was found not to be significant.

Transplanted NPCs were identified by GFP positivity and by clusters of cells that were distinct from the hippocampal cytoarchitecture. NPCs were predominately located at the injection location—surrounding the hippocampal fissure near the molecular layer of the dentate gyrus, without evidence of integration into the granule cell layer (Figure 2(e)).

### 3.3. Injured Animals That Received NPCs Show Improved Performance on NOR as Compared to Injured Animals

**Figure 3:**
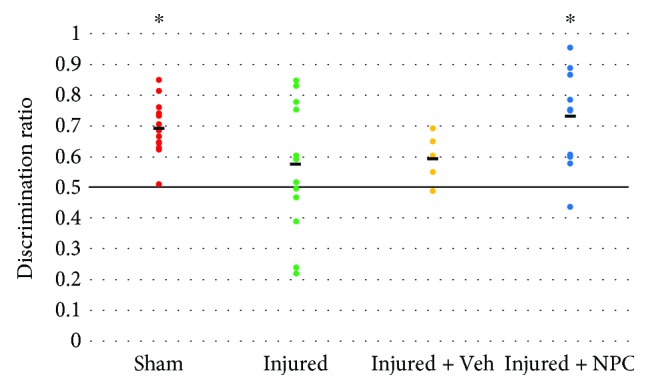
Both injured and injured animals that received vehicle performed poorly on NOR with discrimination ratios that were not significantly different from the test discrimination value of 0.5 (horizontal line; *p* = 0.170 and *p* = 0.114, respectively). Injured animals that received NPCs performed similar to sham animals and spent significantly more time with the novel object in comparison to the familiar object (both *p* < 0.01 versus test value of 0.5, denoted by ∗). Bold lines represent the group mean values.

Sham animals (*n* = 15) were able to adequately distinguish the novel object from the familiar object with a mean discrimination ratio of 0.69, which was significantly different than the test value of 0.5 (*p* < 0.001). In contrast, injured animals (*n* = 15) performed poorly on NOR, with a mean discrimination ratio of 0.57, which was not significantly different from the test value (*p* = 0.170). Injured animals that received vehicle (*n* = 8) had a mean discrimination ratio of 0.59, which also was not significantly different from the test value (*p* = 0.114). Finally, injured animals that received an injection of NPCs showed statistically superior performance (*n* = 11; mean discrimination ratio 0.73) as compared to the test value of 0.5 (*p* = 0.001) (Figure 3).

### 3.4. Implantation of Stem Cells Eliminates the Increase in New Neuron Production following Injury

**Figure 4:**
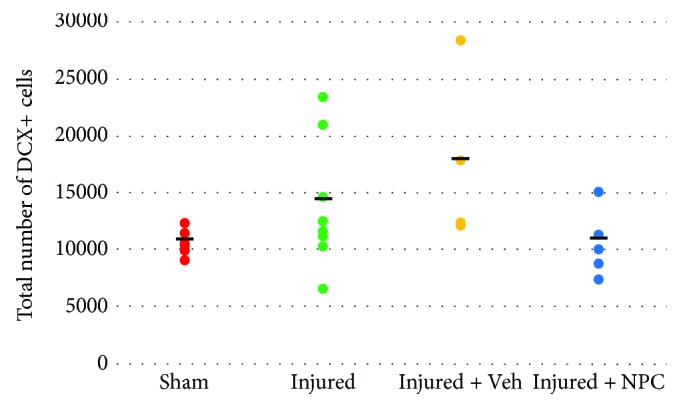
Implantation of NPCs prevents the increase in the number of dentate gyrus DCX+ cells. Post hoc analysis showed no difference in the number of DCX-positive cells between injured and injured animals that received vehicle (*p* = 0.438). While the injured group alone did not show a significant difference from sham (*p* = 0.316), the injured + vehicle group and the combination of these two injury groups showed statistically significant differences (*p* = 0.033 and *p* = 0.050, resp.) with sham. The injured + vehicle group also showed significantly greater DCX-positive cells (*p* = 0.044) than the injured animals that received NPCs. Injured animals receiving NPCs did not have statistically different numbers from sham (*p* > 0.9) or injured animals alone (*p* = 0.377). Bold lines represent the group mean values.

Between-group analysis showed a difference in the total number of hippocampal dentate gyrus DCX-positive cells (*F*[3,26] = 3.793, *p* = 0.022). Post hoc analysis showed no difference in the number of DCX-positive cells between injured and injured animals that received vehicle (*p* = 0.438). While the injured group alone (*n* = 10) did not show a significant difference from sham (*p* = 0.316), the injured + vehicle group (*n* = 5) and the combination of these two injury groups showed statistically significant differences (*p* = 0.033 and *p* = 0.050, resp.) with sham (*n* = 8). The injured + vehicle group also showed significantly greater DCX-positive cells (*p* = 0.044) than the injured animals that received NPCs (*n* = 7). Injured animals receiving NPCs did not have statistically different numbers from sham (*p* > 0.9) or injured animals alone (*p* = 0.377) (Figure 4).

### 3.5. Dentate Gyrus of Injured Animals Contains Immature Neurons with Abnormal Features

**Figure 5:**
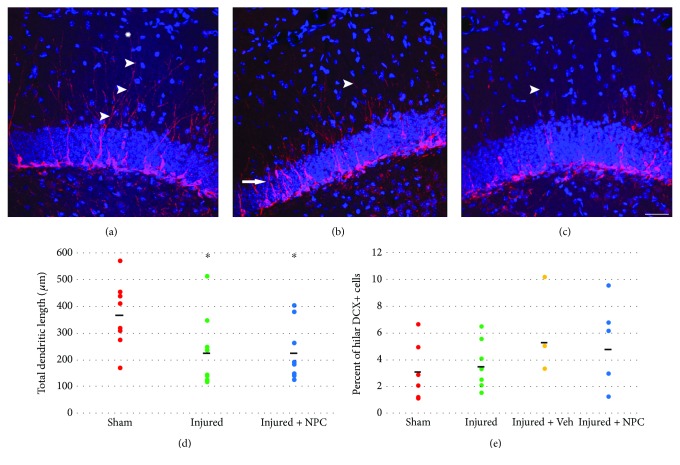
Injured animals show an increase in immature neurons with abnormal features. (a) Sham animals show DCX+ cells with branching dendrites that extend well into molecular layer. Arrowhead follows a dendrite that spans the majority of the molecular layer, and longer dendrites are also present (asterisk). (b) Injured animals show DCX reactivity that demonstrates large clusters of immature cells, some inappropriately located in the middle and upper granule cell layer (arrow). The DCX+ cells show less extensive branching into the molecular layer (arrowhead). (c) Smaller DCX+ cells with fewer branches and shorter dendrites (arrowhead) are also seen in injured animals that received NPCs. (a, b, c) Red = DCX antibody reactivity, blue = DAPI nuclear stain, and scale bar = 100 *μ*m. (d) Additionally, the overall dendritic length is significantly shorter for DCX+ cells in injured animals (post hoc *p* = 0.021) and those that received NPCs (post hoc *p* = 0.020). (e) DCX+ cells were located abnormally in the hilus in all animal groups but there was no significant difference between groups (*p* = 0.263). Bold lines represent the group mean values.

Sham animals have dentate gyrus immature neurons that demonstrated dendrites extending well into the molecular layer with complex branching (Figure 5(a)). This pattern of dendritic branching was not demonstrated in the majority of DCX-positive cells seen in injured animals and injured animals that received NPCs (Figures 5(b) and 5(c)). Analysis of the total dendritic length of DCX-positive cells, including the sum length of all branches on each cell, showed a significant between-group variance (*F* [2, 30] = 5.427, *p* = 0.010). Specifically, dendritic length was significantly longer in sham animals as compared to injured (post hoc *p* = 0.021) or injured animals that received NPCs (post hoc *p* = 0.020) (injured + vehicle not available; Figure 5(d)). In addition, animals from all groups had DCX-positive cells abnormally located in the hilus (Figure 5(e)), but there were no significant differences in the proportion of DCX-positive cells located in this region (*F*[3,26] = 1.406, *p* = 0.263).

## 4. Discussion

We have shown that after a moderate-to-severe LFPI, mice display cognitive improvement after implantation of NPCs that is not associated with an increase in neuron number or with advanced morphological maturation of endogenous injury-induced immature neurons.

### 4.1. Relevance of the Lateral Fluid Percussion Injury Model

Lateral fluid percussion injury is a well-established animal model of TBI [13, 46]. While the majority of LFPI studies have been undertaken in rats, the model has also been adapted for mice [30]. We chose mice for this study because transplantation of mouse-derived NPCs into mouse brain eliminated the need for immunosuppressant therapy that could potentially confound our results and additionally allows for future studies of gene-based perturbations using available transgenic animals.

Lateral FPI elicits focal and diffuse damage including cortical and subcortical (white matter) hemorrhage, blood-brain barrier breakdown, and diffuse axonal pathology; thus, it mimics human TBI [13, 47, 48]. With LFPI, there is documented hippocampal neuronal death, impaired long-term potentiation [13], and altered calcium levels [14]. The LFPI is our preferred model for studying TBI as it provides appropriate hippocampal injury without gross tissue cavitation.

We produced LFPI in mice with RRTs consistent with previously published results. Studies utilizing LFPI in mice have defined mild injury as RRT between 2 and 4 minutes [49, 50], moderate injuries as RRT between 200 and 600 seconds, and severe injuries as those with RRT ≥ 540 seconds [51–55]. Thus, our RRT range of 260–660 seconds (~ 4–11 minutes) is appropriate for the goal of a moderate-to-severe injury. Some groups also report tonic posturing, a fencing response, as an additional validation of moderate injury severity [55, 56]. We acknowledge similar responses in our animals; however, these were not explicitly recorded so the data are not presented here.

### 4.2. Transplanted NPCs Enhance Cognitive Function after LFPI

Many studies show functional improvements after stem cell transplantation; however, the mechanism underlying the cognitive improvement with cell transplantation is not known. We transplanted NPCs into the hippocampal fissure, with the goal of detecting whether direct effects on the hippocampus influence cognitive recovery. Other studies of stem cell transplantation have used a variety of transplant locations including striatum [57] and perilesional cortex [5, 9, 58] and demonstrate improvements in hippocampal cognitive tasks such as Morris water maze. In our study, we evaluated animals 1 week after transplantation. One week is too early to allow for transplanted cells to incorporate into the hippocampal circuitry and, as expected, we did not observe evidence of cell migration or integration. We found transplanted cells located only in the vicinity of the implant location near the hippocampal fissure and did not see graft cells in the dentate gyrus.

Despite the lack of transplanted cell migration and incorporation into the dentate gyrus, we found performance enhancement on novel object recognition (Figure 3). NOR is a hippocampal-dependent test of visual object recognition memory [44, 45]. Other groups exploring cognition after experimental TBI have noted injury-induced deficits in NOR performance [42, 59]. We found poor performance on NOR after injury and statistically significant cognitive improvement one week after NPC transplantation. This suggests that transplanted cell integration is not necessary for cognitive enhancement in this injury model.

Others have suggested that growth factors and neurotrophins enhance graft survival, integration, and hence functional improvement. Early improvements in learning and memory after cell transplantation posit glial-derived neurotrophic factor as a critical supporter of graft survival and neuronal differentiation [9, 10]. Blaya and colleagues [5] injected NPCs secreting a synthetic multineurotrophin into the perilesional cortex of rats one week after LFPI. They demonstrated improvement in graft survival and neuronal differentiation at 5 weeks posttransplantation that was neurotrophin secretion dependent. However, they found that spatial learning enhancement was independent of neurotrophin secretion. Our findings support the previous literature, as we found cognitive improvement early after cell transplantation, which implies a mechanism of action distinct from graft survival and integration.

### 4.3. Increased Neurogenesis Does Not Underlie Cognitive Improvement after Cell Implantation

We evaluated immature neuron production after TBI and changes at one week postcell transplantation. We show overall increases in immature neuron number after LFPI. Simple comparison of all of our injured animals that did not receive NPCs versus sham shows a significant increase in DCX numbers after injury (*p* = 0.050) that is consistent with similar increases reported by others after experimental TBI [21–26]. Specifically, studies looking at the response of endogenous progenitor cells to TBI have demonstrated an increase in DCX cells at seven days after either LFPI or controlled cortical impact in C57BL/6 mice [23, 26].

Further, our multiple comparison analysis for this study suggests that injured animals that received an injection of vehicle did not show a statistically significant difference in DCX number as compared to injured animals without injection, arguing against an independent effect of vehicle alone on neurogenesis. Notably, the injured animals that received NPCs did not show an increase in DCX cell number as compared to the sham group (Figure 4).

New neurons generated after experimental TBI have been shown to integrate into the hippocampal circuitry [60], and thus the importance of adult neurogenesis for the restoration of cognition is a reasonable question. Others have evaluated ablation of neurogenesis after experimental TBI and have shown that cognitive recovery one month after injury is diminished when adult neurogenesis is abolished [27, 28]. Our animals with transplanted NPCs showed improved NOR performance without evidence of additional new neuron production (Figure 3). Hence, the overall process of neurogenesis is important for cognitive improvement after TBI, but the timing, integration, and role of these new neurons remain unclear.

We additionally showed that new neurons in the injured dentate gyrus are likely abnormal in comparison to their sham counterparts. Abnormal cell clusters, fewer dendritic branches, and a shorter dendritic tree length reflect this notion (Figure 5). In other injury systems, such as epilepsy models, there is evidence of abnormal neurogenesis with injury. Shapiro and Ribak [61] have shown that the injury-induced increase in new neuron production leads to DCX labeled cell clusters that lack the normal one-to-one association with astrocytes and harbors persistent basal dendrites. Parent and colleagues [62] demonstrated that seizures generate increases in new neurons that show ectopic hilar migration. Recently, it has been demonstrated that TBI-induced neurogenesis may show similar signs of abnormal neurogenesis, such as inappropriate maturation, migration, and integration with hippocampal circuitry [63, 64].

We did not find a difference in the proportion of abnormally located hilar DCX-positive neurons, but we demonstrate shorter dendritic lengths in immature neurons after injury. Animals that received NPCs showed immature neurons with abnormal characteristics but there were overall fewer new neurons. This suggests that there may be a “goldilocks effect” with respect to adult neurogenesis; too many new neurons may be just as detrimental as too few. Our statistical analysis on dendritic lengths is limited by the possibility of a clustering effect related to repeated measures performed within each mouse (5 cells analyzed for mice in each group). A more robust multilayered analysis will be required to confirm the validity of our findings. If confirmed to be true, cognitive performance would be at its best when the number of appropriately integrated new neurons correctly matches the intrinsic circuitry. Thus, the exact mechanism by which NPC transplantation improves cognition is still largely unknown but could be related to restoration of the homeostasis of endogenous neurogenesis.

We have shown that cognitive improvement is not dependent on the integration of transplanted cells—as we did not observe qualitative signs of integration at the one-week posttransplantation time that is too short to expect graft cell incorporation into existing circuitry. Secondly, NPCs did not produce any further increases in immature neuron production; thus, the mechanism for cognitive improvement does not involve additional cell generation. Lastly, we showed that LFPI-induced new neurons are abnormal and that NPC transplantation did not alter the abnormal characteristics of these immature cells.

## 5. Conclusions

We demonstrated that after a moderate-to-severe experimental TBI, mice demonstrate deficits in cognitive function and an increase in adult neurogenesis. Improved cognition after NPC transplantation did not occur via graft cell integration into the endogenous circuitry, but perhaps through modulation of the timing, number, and integration of abnormal endogenous new neurons. Further understanding of injury-induced aberrant neurogenesis may yield an important target for future therapeutic intervention.

## Abbreviations

DCX: Doublecortin
DPI: Days postinjury
ES: Embryonic stem cells
FPI: Fluid percussion injury
GFAP: Glial fibrillary acidic protein
GFP: Green fluorescent protein
NOR: Novel object recognition
NPC: Neural progenitor cells
PBS: Phosphate-buffered solution
rpm: Rotations per minute
RRT: Righting reflex time
TBI: Traumatic brain injury.
